# Supplementary material for: Combining Essential Oils with Each Other and with Clotrimazole Prevents the Formation of Candida Biofilms and Eradicates Mature Biofilms
Source: Pharmaceutics. 2022 Sep 5;14(9):1872. doi: 10.3390/pharmaceutics14091872 (PMC9503487; doi:10.3390/pharmaceutics14091872)
Supplement: Supplementary file 1 [file pharmaceutics-14-01872-s001.zip › pharmaceutics-1810897-supplementary.pdf]

pharmaceutics

MDPI

Supplementary Materials: Combining Essential Oils with Each Other and with Clotrimazole Prevents the Formation of *Candida* Biofilms and Eradicates Mature Biofilms

Rafael Alves da Silva, Nagela Bernadelli Sousa Silva, Carlos Henrique Gomes Martins, Regina Helena Pires, Denise Von Dolinger de Brito Röder and Reginaldo dos Santos Pedroso

Table S1. Combinations between OE-OE and OE-clotrimazole in *Candida* species.

Species	Compound "A"	Compound "B"	Isolated MIC "A"*	Isolated MIC "B"*	MIC "A" combined**	MIC "B" combined**	FICI	Result
C. albicans ATCC 90028	*L. cubeba*	*M. alternifolia*	1000	4000	500	2000	1	Additive
C. albicans ATCC 90028	*C. limon*	Clotrimazole	2000	0,25	4000	1	6	Antagonism
C. albicans ATCC 90028	*C. sempervirens*	*C. limon*	2000	2000	4000	4000	4	Indifference
C. albicans ATCC 90028	*C. sempervirens*	*L. cubeba*	2000	1000	1000	1000	1,5	Indifference
C. albicans ATCC 90028	*C. sempervirens*	*M. alternifolia*	2000	4000	4000	4000	3	Indifference
C. albicans ATCC 90028	*L. cubeba*	*C. limon*	1000	2000	2000	2000	3	Indifference
C. albicans ATCC 90028	*C. limon*	*M. alternifolia*	2000	4000	4000	4000	3	Indifference
C. albicans ATCC 90028	*C. sempervirens*	Clotrimazole	2000	0,25	250	0,5	2,125	Indifference
C. albicans ATCC 90028	*L. cubeba*	Clotrimazole	1000	0,25	250	0,5	2,25	Indifference
C. albicans ATCC 90028	*M. alternifolia*	Clotrimazole	4000	0,25	250	0,063	0,3145	Synergism
C. albicans SV01	*C. sempervirens*	*L. cubeba*	4000	2000	2000	1000	1	Additive
C. albicans SV01	*C. sempervirens*	*M. alternifolia*	4000	4000	2000	1000	0,75	Additive
C. albicans SV01	*C. sempervirens*	Clotrimazole	4000	0,125	500	0,063	0,629	Additive
C. albicans SV01	*M. alternifolia*	Clotrimazole	4000	0,125	250	0,063	0,5665	Additive

C. albicans SV01	*C. sempervirens*	*C. limon*	4000	4000	4000	4000	2	Indifference
C. albicans SV01	*L. cubeba*	*C. limon*	2000	4000	2000	2000	1,5	Indifference
C. albicans SV01	*C. limon*	*M. alternifolia*	4000	4000	4000	4000	2	Indifference
C. albicans SV01	*C. limon*	Clotrimazole	4000	0,125	4000	0,25	3	Indifference
C. albicans SV01	*L. cubeba*	Clotrimazole	2000	0,125	2000	0,25	3	Indifference
C. albicans SV01	*L. cubeba*	*M. alternifolia*	2000	4000	500	1000	0,5	Synergism
C. glabrata ATCC 2001	*C. sempervirens*	*L. cubeba*	2000	2000	1000	500	0,75	Additive
C. glabrata ATCC 2001	*L. cubeba*	*M. alternifolia*	2000	4000	1000	1000	0,75	Additive
C. glabrata ATCC 2001	*C. limon*	*M. alternifolia*	4000	4000	2000	2000	1	Additive
C. glabrata ATCC 2001	*C. sempervirens*	Clotrimazole	2000	0,015	4000	0,06	6	Antagonism
C. glabrata ATCC 2001	*C. limon*	Clotrimazole	4000	0,015	4000	0,06	5	Antagonism
C. glabrata ATCC 2001	*M. alternifolia*	Clotrimazole	4000	0,015	4000	0,06	5	Antagonism
C. glabrata ATCC 2001	*C. sempervirens*	*C. limon*	2000	4000	2000	250	1,0625	Indifference
C. glabrata ATCC 2001	*C. sempervirens*	*M. alternifolia*	2000	4000	2000	250	1,0625	Indifference
C. glabrata ATCC 2001	*L. cubeba*	Clotrimazole	2000	0,015	2000	0,015	2	Indifference
C. glabrata ATCC 2001	*L. cubeba*	*C. limon*	2000	4000	500	1000	0,5	Synergism
C. glabrata SV 02	*C. sempervirens*	*M. alternifolia*	1000	4000	500	1000	0,75	Additive
C. glabrata SV 02	*L. cubeba*	*C. limon*	2000	4000	500	2000	0,75	Additive
C. glabrata SV 02	*C. sempervirens*	*C. limon*	1000	4000	4000	4000	5	Antagonism
C. glabrata SV 02	*C. limon*	Clotrimazole	4000	0,25	4000	1	5	Antagonism
C. glabrata SV 02	*M. alternifolia*	Clotrimazole	4000	0,25	4000	1	5	Antagonism
C. glabrata SV 02	*C. sempervirens*	*L. cubeba*	1000	2000	1000	1000	1,5	Indifference
C. glabrata SV 02	*C. sempervirens*	Clotrimazole	1000	0,25	1000	0,5	3	Indifference
C. glabrata SV 02	*L. cubeba*	Clotrimazole	2000	0,25	2000	0,5	3	Indifference
C. glabrata SV 02	*L. cubeba*	*M. alternifolia*	2000	4000	250	1000	0,375	Synergism
C. glabrata SV 02	*C. limon*	*M. alternifolia*	4000	4000	1000	1000	0,5	Synergism
C. krusei ATCC 6258	*C. sempervirens*	*L. cubeba*	2000	1000	500	500	0,75	Additive
C. krusei ATCC 6258	*C. sempervirens*	*M. alternifolia*	2000	4000	1000	1000	0,75	Additive

C. krusei ATCC 6258	*M. alternifolia*	Clotrimazole	4000	0,5	2000	0,125	0,75	Additive
C. krusei ATCC 6258	*C. limon*	Clotrimazole	4000	0,5	4000	2	5	Antagonism
C. krusei ATCC 6258	*L. cubeba*	*M. alternifolia*	1000	4000	2000	250	2,0625	Indifference
C. krusei ATCC 6258	*C. sempervirens*	Clotrimazole	2000	0,5	2000	0,125	1,25	Indifference
C. krusei ATCC 6258	*L. cubeba*	*C. limon*	1000	0,5	2000	0,25	2,5	Indifference
C. krusei ATCC 6258	*L. cubeba*	Clotrimazole	1000	0,5	2000	0,25	2,5	Indifference
C. krusei ATCC 6258	*C. sempervirens*	*C. limon*	4000	4000	1000	250	0,3125	Synergism
C. krusei ATCC 6258	*C. limon*	*M. alternifolia*	4000	4000	1000	1000	0,5	Synergism
C. krusei SV 03	*C. sempervirens*	Clotrimazole	500	0,5	2000	0,125	4,25	Antagonism
C. krusei SV 03	*C. limon*	Clotrimazole	1000	0,5	4000	2	8	Antagonism
C. krusei SV 03	*C. sempervirens*	*C. limon*	500	1000	1000	1000	3	Indifference
C. krusei SV 03	*C. sempervirens*	*L. cubeba*	500	500	500	250	1,5	Indifference
C. krusei SV 03	*C. sempervirens*	*M. alternifolia*	500	2000	1000	250	2,125	Indifference
C. krusei SV 03	*L. cubeba*	*C. limon*	500	1000	1000	250	2,25	Indifference
C. krusei SV 03	*L. cubeba*	*M. alternifolia*	500	2000	1000	250	2,125	Indifference
C. krusei SV 03	*C. limon*	*M. alternifolia*	1000	2000	2000	2000	3	Indifference
C. krusei SV 03	*L. cubeba*	Clotrimazole	500	0,5	500	0,5	2	Indifference
C. krusei SV 03	*M. alternifolia*	Clotrimazole	2000	0,5	2000	0,125	1,25	Indifference
C. parapsilosis ATCC 22019	*L. cubeba*	*M. alternifolia*	1000	4000	500	2000	1	Additive
C. parapsilosis ATCC 22019	*C. sempervirens*	*C. limon*	4000	1000	4000	4000	5	Antagonism
C. parapsilosis ATCC 22019	*C. limon*	*M. alternifolia*	1000	4000	4000	4000	5	Antagonism
C. parapsilosis ATCC 22019	*L. cubeba*	Clotrimazole	1000	0,25	4000	1	8	Antagonism
C. parapsilosis ATCC 22019	*C. sempervirens*	*L. cubeba*	4000	1000	1000	1000	1,25	Indifference
C. parapsilosis ATCC 22019	*C. sempervirens*	*M. alternifolia*	4000	4000	4000	4000	2	Indifference
C. parapsilosis ATCC 22019	*L. cubeba*	*C. limon*	1000	1000	1000	1000	2	Indifference
C. parapsilosis ATCC 22019	*C. sempervirens*	Clotrimazole	4000	0,25	250	0,5	2,0625	Indifference
C. parapsilosis ATCC 22019	*M. alternifolia*	Clotrimazole	4000	0,25	250	0,25	1,0625	Indifference
C. parapsilosis ATCC 22019	*C. limon*	Clotrimazole	1000	0,25	1000	0,125	1,5	Indifference

C. parapsilosis SV 04	*C. sempervirens*	*C. limon*	4000	4000	2000	250	0,5625	Additive
C. parapsilosis SV 04	*C. sempervirens*	*L. cubeba*	4000	2000	1000	1000	0,75	Additive
C. parapsilosis SV 04	*C. sempervirens*	*M. alternifolia*	4000	4000	2000	250	0,5625	Additive
C. parapsilosis SV 04	*L. cubeba*	*C. limon*	2000	4000	2000	2000	1,5	Indifference
C. parapsilosis SV 04	*L. cubeba*	*M. alternifolia*	2000	4000	2000	250	1,0625	Indifference
C. parapsilosis SV 04	*C. limon*	*M. alternifolia*	4000	4000	4000	4000	2	Indifference
C. parapsilosis SV 04	*L. cubeba*	Clotrimazole	2000	0,125	2000	0,25	3	Indifference
C. parapsilosis SV 04	*C. sempervirens*	Clotrimazole	4000	0,125	250	0,032	0,3185	Synergism
C. parapsilosis SV 110	*C. limon*	Clotrimazole	4000	0,125	2000	0,125	1,5	Indifference
C. parapsilosis SV 110	*M. alternifolia*	Clotrimazole	4000	0,125	2000	0,125	1,5	Indifference

MIC: µg/mL (able to reduce by ≥90% optical density (OD) compared to control free of EO and clotrimazole). *Isolated MIC: Only 1 substance (EO or clotrimazole). ** MIC Combined: MIC of the combination (EO-EO or EO-clotrimazole). FICI: fractional inhibitory concentration index.
